# Motivators and barriers to uptake of post-operative voluntary medical male circumcision follow-up in Yala division, Siaya County, Kenya

**DOI:** 10.11604/pamj.supp.2016.25.2.9369

**Published:** 2016-11-26

**Authors:** Bonface Abunah, Rueben Onkoba, Josephat Nyagero, Samuel Muhula, Edward Omondi, Bernard Guyah, Gregory Barnabas Omondi

**Affiliations:** 1 Amref Health Africa in Kenya, Monitoring, Evaluation and Research, Kenya; 2 Maseno University, School of Public Health and Community Development, Kenya; 3 KEMRI, Centre for Global Health Research, Kenya

**Keywords:** Barriers, motivators, voluntary medical male circumcision, post-operative follow-up

## Abstract

**Introduction:**

Follow-up visits are recommended to all voluntary medical male circumcision clients (VMMC), however, adherence is variable. High lost-to-follow-up cases limit knowledge about clinical status of clients and adverse events. This study sought to establish Motivators and Barriers to the Uptake of VMMC post-operative follow-up services in Siaya County, Kenya.

**Methods:**

277 clients from five VMMC sites in Yala were recruited immediately post-operation to participate in a telephone interview between the 21st and 31st day post-surgery during which a semi-structured questionnaire was administered. Descriptive and inferential statistics was used to analyse quantitative information using SPSS while responses from open ended questions were grouped into themes, sieved out, coded and analyzed.

**Results:**

137(49.5%) of the 277 participants utilized the follow-up services. Health education (31.4%) and emergency reviews/adverse events (24.1%) were the main motivation for returning for follow-up while occupational and other engagements (29.7%) and presumption of healing (24.6%) were the main barriers. Type of facility attended (p=0.0173), satisfaction with the discharge process (p=0.0150) and residency in Yala (p<0.001) were statistically significant to the respondents’ return for follow-up. 85(62.0%) of the participants returned on the 7th day, 9(6.6%) returned after 7 days, and 43(31.4%) returned before 7 days.

**Conclusion:**

VMMC health education should include and emphasize the benefits of follow-up care to the clients and the providers should address the barriers to accessing follow-up services. Our results will inform the programme on areas identified to improve care for VMMC clients and reduce subsequent lost-to-follow-up cases.

## Introduction

After decades of research, innovations and implementation of interventions, the path to zero new HIV infections is becoming clearer. Implementation of male circumcision programs at large scale has a larger impact on the human-immunodeficiency virus (HIV) epidemic. Three randomized controlled trials showed that male circumcision (MC) reduces the risk of heterosexual transmission of HIV by approximately 50-60% [1–3]. Following this, the World Health Organization (WHO) and UNAIDS, in March 2007, issued guidelines urging countries with low male circumcision and high HIV prevalence to include male circumcision in their comprehensive HIV prevention programmes [4]. Scaling up of VMMC to over 80% of men in reproductive age across the world would prevent acquisition of millions of new infections and would save a lot of money that would have been used in care and treatment [5]. Kenya’s Ministry of Health adopted a new policy on male circumcision in 2007 as a response to the call by WHO and UNAIDS based on evidence from the trials. The policy recognized the importance of male circumcision in preventing HIV and other sexually transmitted infections in men and promoted MC alongside other HIV prevention strategies [6]. Male circumcision was therefore added by National AIDS and STI Control Programme (NASCOP) as one additional HIV prevention strategy that would be rolled out, especially in areas of high HIV and STDs prevalence. The policy aimed at increasing demand for MC and building capacity of health systems to provide safe MC services.

The Male Circumcision Consortium together with the Kenyan Government and other local partners worked closely in the development and implementation of the National male circumcision strategy in 2008 with the initial roll-out in former Nyanza Province [7]. Male circumcision provision has a minimum package which includes: HIV testing and counseling (HTC); provision and promotion of male and female condoms; STI screening, diagnosis and treatment; male circumcision surgical procedure; counseling on risk reduction and safer sex; post-operative follow-ups and referrals. Client follow-ups and referrals contribute to the expanded VMMC package. The VMMC programme has set out targets for each element to be met by the service providers. Most often the targets for some elements, especially post-operative follow-ups, are rarely met even though the services are free at all stages of client care. Post-operative follow-up refers to the care offered to clients by health care worker upon return to the health facility after circumcision. This is the visit where the wound healing status is assessed and treatment is offered of any complication/adverse event identified. The client-provider interaction during the visit offers opportunity for reassurance, counselling, reinforcement of key messages, provision of condoms and testing services. The VMMC guideline recommends a 7th day follow-up visit post circumcision for monitoring outcomes, identifying adverse events and reinforcing key messages on abstinence and risk reduction. However, a large proportion of men fail to return making post-operative follow-up a challenge in the implementation of male circumcision programme.

The programme has a goal of following-up and reviewing on day 7 at least 60% of the VMMC clients served, but the numbers of men returning for follow-up are considerably lower than desired. The fact that a good proportion of men who underwent the services were not coming back for post-operative follow-up raised concern about their well-being. Little has been done to assess the determinants of uptake of scaled-up male circumcision post-operative follow-up. Muula et al. while looking at the complications of male circumcision realized that data was insufficient to support and estimate prevalence measurements [8]. Penile amputation, hematoma and even death are some of the documented serious complications and adverse events following male circumcision, however, most of the complications are attributed to poor surgical skills and lack of proper monitoring [9–11]. Bailey studied 1007 males in Western Kenya and found that 25% of circumcised males experienced an adverse event (35% in traditional circumcisions, 18% in clinical settings) [9], his findings showed that complications and adverse events are as a result of inappropriate training of clinical staff, inadequate follow-up of circumcised clients and inappropriate clinical settings for VMMC. There was a need to probe reasons for clients’ failure to return for follow-ups. The primary objective of this study, therefore, was to establish the motivators and barriers to the uptake of VMMC follow-up and establish the strongest and weakest areas in VMMC service delivery. The main outcomes of interest were the determinants of uptake of post-operative follow-up and the ratings of the components of service delivery.

## Methods

**Study area:** the study was conducted in Yala Division, Siaya County at five health facilities namely Yala Sub-District Hospital, Marenyo Health Centre, Malanga Health Centre, Ramula Health Centre, and Nyawara Health Centre where VMMC has been conducted since 2008.

**Study population:** the study population consisted of men coming to Yala Division for VMMC services at the five public health facilities. They were invited to participate in the study and only those who consented were included.

**Study design:**

**Sampling design and size:** power analysis was used to determine the sample size with comparison being based on the type of the health facility (hospital versus Health Centres). A sample of 277 clients was studied, 56 clients from subgroup A (clients attended to at the Hospital - Yala SDH) and 221 clients from subgroup B (clients attended to at the four Health Centres). Complete census method of sampling was used to recruit the 277 male circumcision clients from the five facilities. The clients were recruited one after the other until the desired number was reached. Inclusion criteria: clients who were studied met the following

**criteria:** Circumcised at a participating VMMC site during the study recruitment period; Provided written consent to participate; Willingness to share information on circumcision experience; Aged 18 years and above.

**Data collection instruments:** the study participants were recruited on the day they received VMMC services after administration of informed consents. Information on participants’ location was also collected. A phone interview was then conducted by an experienced research nurse to the recruited participants between the 21st and 31st day following their VMMC procedure using a semi-structured questionnaire. It included both positively and negatively worded and open-ended questions to minimize potential bias that would have occurred from clustering of responses on one side of the scale.

**Data analysis:** data was collected using semi-structured questionnaires with both closed and open-ended questions. A data entry program was designed in SPSS software (version 18) for all questions and data entered with the serial numbers as unique identifiers. Analysis was then done using descriptive and inferential statistics. Responses from open-ended questions were grouped into themes, sieved out, coded and analyzed based on the study objectives. Statistical significance level was set at p<0.05 and emerging trends and relationships presented in tables.

**Ethical considerations:** ethical approval and clearance was obtained from the Maseno University Ethics Review Committee. Respondent identity was filed under code numbers and the data forms and any other materials for this study had no provision for names. All personal information collected was kept confidential. Only the study personnel had access to the information records and personal identifiers if any.

## Results

### Study population

Of the 1853 VMMC clients who were invited to participate in the study, 277 were eligible and enrolled as study participants. Almost half 125(45.1%) were between the ages of 18-25 years; 151(54.5%) were residents of Yala; 110(39.7%) had completed secondary education; 153(55.2%) had a child and 104(37.6%) were unemployed (Table 1).

**Table 1:** Characteristics of participants enrolled

Characteristics of study participants					
			Returned For Follow-up		
		Total (N=277)	Yes (n=137)	No (n=140)	P Value
**Demographic characteristics and variables**					
**Age in Years**					
	18-25	125(45.1%)	55(44%)	70(56%)	0.19
	26-35	95(34.3%)	47(49%)	48(51%)	
	36-45	25(9.1%)	13(52%)	12(48%)	
	46-55	22(7.9%)	16(73%)	6(27%)	
	56-65	8(2.9%)	5(63%)	3(37%)	
	Over 65	2(0.7%)	1(50%)	1(50%)	
**Education Level**					
	No formal educ.	32(11.5%)	16(50%)	16(50%)	0.07
	Primary	60(21.7%)	36(60%)	24(40%)	
	Secondary	110(39.7%)	44(40%)	66(60%)	
	college	51(18.4%)	30(59%)	21(41%)	
	University	24(8.7%)	11(46%)	13(54%)	
**Employment Status**					
	Permanent	42(15.1%)	24(57%)	18(43%)	0.5
	Temporary	61(22.0%)	32(52%)	29(48%)	
	Self employed	70(25.3%)	35(50%)	35(50%)	
	Unemployed	104(37.6%)	46(44%)	58(56%)	
**Child Status**					
	Has children	153(55.2%)	82(54%)	71(46%)	0.15
	No children	124(44.8%)	55(44%)	69(56%)	
**Permanent resident of Yala**					
	Yes	151(54.5%)	90(60%)	61(40%)	<0.0001
	No	126(45.5%)	47(37%)	79(63%)	
**Health Facility**					
	Malanga(OPD)	24(8.7%)	14(58%)	10(42%)	0.017
	Marenyo(OPD)	87(31.4%)	41(47%)	46(53%)	
	Nyawara(OPD)	91(32.8%)	46(51%)	45(49%)	
	Ramula(OPD)	19(6.9%)	3(16%)	16(84%)	
	Yala SDH(IPD)	56(20.2)	33(59%)	23(41%)	

### Post-operative follow-up rates and timeliness

137(49.5%) of the respondents returned for post-operative follow-ups at the service sites and 34(24.8%) of those who returned never found service provision teams at the sites. In terms of their timeliness to return for visits, 85(62.0%) returned on the scheduled day (on 7th day), 9(6.6%) returned after 7 days, and 43(31.4%) returned before 7 days. Majority of those who returned for the visits on time (that is on the 7th day) were from the ages of 26-35 years (35.3%), had attained secondary school education (35.3%), were married (63.5%), residents of Yala (67.1%) and in temporary employment (27.1%). Considering the follow-up rates per facility Yala SDH recorded the highest rate by reviewing 58.9% of their clients. Malanga, Nyawara, Marenyo and Ramula Health Centres reviewed 58.3%, 50.6%, 47.1% and 15.8% respectively, giving an overall follow-up rate of 49.5%. Yala Sub-District Hospital, an in-patient facility reviewed 58.9% of their clients while out-patient facilities (Health Centres) reviewed 47.1% of their clients. This showed that respondents preferred revisiting the hospital setup as opposed to the Health Centre setup. It is worth noting that neither the individual facilities nor the five of them together met the required guideline of reviewing at least 60% of the served clients. 99(35.7%) respondents visited other practitioners for care and 63(63.6%) of them paid for those services, contrary to the policy guidelines for the roll-out of VMMC which states that care should be free at all stages.

### Motivators for VMMC Post-operative follow-up

Out of the 277 respondents in this survey, 137(49.5%) returned for follow-ups. This study sought to establish the reasons as to why some clients returned for the scheduled visits and others did not through open ended questions. 31.4% of those who returned mentioned education/instructions given during counselling as the reason for their return while 24.1% mentioned the occurrence of an adverse event as a reason (Table 2).

**Table 2:** Motivators and barriers to post-operative follow-ups

Reasons for Return for Follow-up	Proportion (%)	Reasons for Non-return for follow-up	Proportion (%)
Education/instruction during counseling	31.4	Occupational and other engagements	29.7
Emergency reviews/Adverse events	24.1	Presumption of healing	24.6
Medication/Bandage removal	19.7	Lack of free transport, Financial Problems, Poor Weather	20.0
Free and efficient services	12.4	Lack of Post-Op care information and fear of pain	15.0
Fear of ill health	12.4	Inconsistency of teams, Nature of service providers	4.3
		Private consultations	3.6
		Distorted body image	1.4
		Non-Response	1.4
Total	100.0	Total	100.0

**Education/instruction during counselling and surgery:** three in every ten (31.4%) of the respondents returned as a result of the emphasis for routine check-up on 7th day during the counselling sessions and after surgery. There were those who returned because they were reminded by client information sheets and appointment cards given during counselling.

**Emergency reviews and adverse events:** slightly less than a quarter (24.1%) of the respondents returned because they developed medical emergencies after surgery and thus needed immediate medical attention. The following are cases that returned before seven days and the reasons as to why they came earlier. *“I had so much blood coming out from the bandage and I had so much pain” “The stitches had a problem and needed to be checked as I was bleeding so much on the 3rd day” “On the third day I noticed my penis was swelling and I had severe pain. On contacting the service providers they asked me to return immediately” “I was playing football when I fell down and ruptured the stitches”*

**Medication and bandage removal:** two in every ten (19.7%) of the respondents returned to replenish stock of pain medication while others who couldn’t comfortably remove the bandages on their own came for their removal.

**Free and efficient services:** 12.4% of the respondents were attracted by the free and efficient services offered and the conduct of the attending personnel who were very efficient, friendly and professional. One respondent described the service: *“Male circumcision is provided free of charge, at no cost and the surgical procedure is very fast and efficient”.* Free transportation offered by the organization affected the day 7 follow-ups, more reviews were registered on the days free transportation was offered compared to the days when free transportation was unavailable.

Fear of ill health: 12.4% of the respondents had fear and were uncertain of their current state and came for medical advice and reassurance on their prevailing condition. One participant said *“I came because I was not sure whether my wound is healing or not, I was so scared and all I needed was an assurance from the doctor that everything is okay”,* while another said *“I have never undergone any surgery before and the sight of the wound was traumatizing to me, therefore I came because I feared something might be wrong that needed the doctors attention”.*

### Barriers to VMMC Post-operative Follow-up

Despite the services being free at all stages of client care, 140(50.5%) of the respondents never utilized the follow-up services. Of those who did not return, 29.7% had occupational and other engagements and 24.6% presumably felt they have healed and needed no further care (Table 2).

**Occupational and other engagements (29.7%):** respondents were drawn from a constituency of males with varied characteristics. A greater proportion missed the scheduled appointments because of resuming duties at their work stations, attending to their daily chores, travelling back to schools, colleges, and visiting relatives outside Yala.

**Presumption of healing (24.6%):** out of the ordinary there were respondents who felt they were healing well or had healed without any complication hence saw no need of going back for review as advised during counselling.

**Transportation, financial problems and poor weather (20.0%):** free transportation of clients acted in favor of and against the day 7 follow-up visits. Respondents who were initially picked by the organization vehicles during the service expected the same during reviews and thus missed the appointments for lack of free transportation. Some respondents expressed difficulties with finances and therefore could not reach the far stationed sites while others were disadvantaged by bad weather.

**Lack of post-operative care information and fear of pain (15.0%):** some respondents claimed not to have heard the information during the counseling sessions while others admitted forgetting the scheduled dates due to loss of appointment cards, lack of reminders, and lack of client information sheets. Interestingly there were respondents who missed the appointments for the fear of more pain being inflicted on their penis during the reviews.

**Inconsistency in availability of teams and nature of service providers (4.3%):** there were respondents who missed the teams providing the services at the sites during the scheduled reviews while others had the fear of finding the same teams that attended to them earlier because of dissatisfaction with the services offered or as a result of inclusion of female surgeons in the teams.

**Private consultation (3.6%):** a number of respondents especially those who were non-residents of Yala and/or those who were permanently employed and had resumed duty opted for the services of private practitioners at a fee hence missing the follow-up appointments. A few others also consulted the service providers over the phone for more instructions on wound care and reassurance.

**Distorted body image (1.4%):** this was a major concern especially for the elderly respondents; they skipped the appointments because their worry rested on the distorted walking styles and the fear of being seen by people known to them.

**Non-response (1.4%):** a small proportion of those who did not return for review did not respond to the question.

### Determinants of post-operative follow-up state

On testing the association between the respondent’s return to the health facility for follow-up and other variables, the type of facility attended (p=0.017), satisfaction with the discharge process (p=0.015) and residency in Yala (p<0.001) were statistically significant to the respondents’ return for follow-up. There was no statistical significant difference for returning to the facility versus age group, education attained, employment status, marital status, religion, history of having children, satisfaction with counselling services, theatre services or surgical outcome and efficiency in the running of services.

### Strongest and weakest areas in VMMC service delivery

Table 3 summarizes the major areas identified by the respondents as the strongest and weakest areas in VMMC service delivery. Staff competency (29%) and free, available and efficient services (27%) ranked highest as positive attributes to the service providers while inconsistency of teams (21%) and long waiting time (18%) ranked highest as negative attributes to the operations of the service provision teams.

**Table 3:** Strongest and weakest areas in VMMC service delivery

Strongest areas in service delivery	Proportion (%)	Weakest areas in service delivery	Proportion (%)
Staff competency	29	Inconsistency of teams and transport	21
Free, available and efficient services	27	Long waiting time	18
Educative and Informative counseling	17	Non-response	12
Intense Mobilization	10	All gender teams representations	11
Non-response	10	Ineffective pain management	10
All gender team representations	4	Lack of Operations during week-ends and in all facilities	10
24 hours emergency response	3	Lack of compensation	9
		Mandatory HIV testing	8
		Inadequate refreshment	1
Total	100	Total	100

## Discussion

VMMC has been implemented on a large scale. Thus, it is important for the service providers to monitor the clients throughout the healing period and emphasize the importance of follow-up care, since satisfied clients are the most effective mobilizers for the national VMMC programme. A VMMC programme should promote post-operative review visits irrespective of the client’s status even though clients who experience complications are more likely to come for reviews. The findings of this study showed that the major (45.1%) beneficiaries of VMMC services are aged between 18-25 years. This is in line with the project recommendations of targeting sexually active males for the prevention of acquisition of new HIV infections. The review rate of 49.5% established by this study is way below the 60% minimum set by the programme and this calls for urgent intervention by the members of male circumcision consortium for quality improvement. A greater proportion (31.4%) returned for post-operative review as a result of proper education/instruction during counselling and surgery, this is in line with other studies that showed that communicating effectively with patients, respecting them and engaging them in the provider-patient relationship are some of the strongest determinants of client-perceived quality [12, 13]. Offering educative sessions on health promotion and prevention of diseases makes patients satisfied with care given and influence their recommendations to others [14]. The type of facility attended was a predictor of the state of follow-up. Most clients preferred revisiting the Hospital set up than the Health Centre’s. Several studies have explored the physical environments in healthcare settings, for example, Woodside found that location, equipment, and facility were important factors that hospital patients sought to optimize [15]. In another study by Gotlieb it was reported that the perceptions of patients´ of their hospital rooms could influence their view of hospital quality service provision [16].

The presence of females as service providers acted both as a motivator and a barrier factor, there were respondents who preferred being attended to by female service providers and those who did not. The preference was mostly determined by age and other socio-demographics of the respondents. This finding is in agreement with a study by Amy Herman-Roloff which reported that making male client’s feel shy is the hindrance to having female service providers, coupled with this is the fear of a client having an erection as the female provider touches the penis during inspection. Amy also reported a section of respondents who had no problems with female providers as long as they are certified and they remain anonymous to them and there were a few others who opted for the females as compared to their male counterparts because of their feminine understanding and gentle nature while attending to someone [17]. Another interesting finding of this study was participants’ fear of more pain being inflicted on their penis during the follow-up visits. This finding is in line with those of Mattson and Bailey where pain was identified as a factor that inhibits the uptake of male circumcision alongside other factors such as cultural tradition, bleeding, infection, and cost [18, 19]. The providers should anticipate pain and address the clients concerns during the counselling sessions in order to improve the uptake and adherence to follow-up visits. Some participants reported presumed healing as a reason for not coming back for follow-up care, however, those who had concerns and were worried about their prevailing state returned for follow-up care. It was unclear though to establish whether the presumed healed state and the concerns resulted from the prior client - provider interactions during service delivery. Receipt of advice from care givers promotes the uptake of follow-up care; this finding was reported in a cross-sectional survey where women with history of gestational diabetes mellitus were recruited and followed up for the uptake of glucose testing [20]. Transportation to the hospital and the health center sites was a barrier to accessing follow-up services identified in the study. Participants reported struggling with the long distances to make it to the far located facilities. Other studies have also reported transportation to be a major problem and hindrance to accessing care [21, 22]. A PMTCT study conducted in Thyolo district in Malawi found out that lack of transportation was a hindrance to accessing PMTCT services and a contributor to loss to follow-ups from the PMTCT programme [23]. Efforts should be geared towards increasing the number of care giving points that are in close proximity to the beneficiaries (geographic and population prioritization). The current study established inconsistency in service provision teams’ schedule of operations as 24.8% of clients who returned for reviews missed them, an element that lowered the review rates and impacted on the general satisfaction of clients. From previous studies items related to accessibility, affordability, convenience and availability of healthcare services emerged as the most important predictors of patient perception of care. These findings on sub-dimensions on quality of service support several researchers’ opinions that quality of service is a predictor of patient satisfaction [14, 24, 25].

Availability of free, available and efficient services ranked high as an attribute to good service delivery in this study, several male circumcision acceptability studies also identified cost as a barrier to getting circumcised, or having a son circumcised. Mattson found in a study that 34% of men who reported that they “preferred” not to be circumcised said that they would get the operation if the cost was less than $3.00 [26]. Key measures of importance of healthcare service delivery attributes includes friendliness of hospital staff, knowledge and competence of staff, treating patients with respect, cleanliness and tidiness of the health facility, overall appearance of the staff, guidance and information provided on patients’ health needs, cost of healthcare, privacy and confidentiality, accessibility and availability of healthcare, waiting time before service and time spent with the healthcare provider [24]. The findings of this study revealed that being treated with respect by the service providers, presence of friendly and competent staff, cleanliness of theatres, accessibility and availability of services, provision of health education, waiting time, cost of healthcare, service time and finally adherence to privacy and confidentiality during treatment were the most predictors for the uptake of day 7 post-operative follow-ups. Additional findings in the current study showed that 35.7% of the respondents sought care elsewhere apart from the service provision sites, and 63.6% of them paid fees for those services. This is contrary to what the VMMC policy advocates of free service delivery at all stages of client care but since they opted for private consultation they had to pay for the services. It’s upon the authorities to look into their operations in ensuring that VMMC clients receive free services and monitoring throughout the healing period as other studies have shown that different categories or classes of people report different levels of satisfaction with care given in private and public facilities [12, 27–29].

## Conclusion

VMMC is critical and important strategy for decreasing HIV infections globally. In order to increase uptake and adherence to post-operative follow-up for VMMC clients, programme needs to address barriers and build upon motivators for follow-up care. Our qualitative findings on the barriers and motivators for VMMC post-operative follow-up are key in designing and developing holistic approaches with potentials of improving follow-up uptake.

## References

[1] Auvert B, Taljaard D, Lagarde E, Sobngwi-Tambekou J, Sitta R, Puren A (2005). Randomized, controlled intervention trial of male circumcision for reduction of HIV infection risk: the ANRS 1265 Trial. PLoS Med..

[2] Bailey RC, Moses S, Parker CB, Agot K, Maclean I, Krieger JN, Williams CF, Campbell RT, Ndinya-Achola JO (2007). Male circumcision for HIV prevention in young men in Kisumu, Kenya: a randomized controlled trial. Lancet..

[3] Gray RH, Kigozi G, Serwadda D, Makumbi F, Watya S, Nalugoda F, Kiwanuka N, Moulton LH, Chaudhary MA, Chen MZ, Sewankambo NK, Wabwire-Mangen F, Bacon MC, Williams CF, Opendi P, Reynolds SJ, Laeyendecker O, Quinn TC, Wawer MJ (2007). Male circumcision for HIV prevention in men in Rakai, Uganda: a randomized trial. Lancet..

[4] (2007). WHO/UNAIDS technical consultation on male circumcision and HIV prevention: research implications for policy and programming..

[5] Njeuhmeli E, Forsythe S, Reed J, Opuni M, Bollinger L, Heard N, Castor D, Stover J, Farley T, Menon V, Hankins C (2011). Voluntary Medical male circumcision: Modeling the impact and cost of expanding Male circumcision for HIV prevention in Eastern and Southern Africa. PLOS Med..

[6] Ministry of Medical Services and Ministry of Public Health and Sanitation (2011). Comprehensive National Health Policy Framework. Ministry of Medical Services and Ministry of Public Health and Sanitation, 2011-2030..

[7] Kaiser Daily HIV/AIDS report (2008). http://www.kaisernetwork.org/Daily_reports/rep_index.cfm?DR_ID=55734.

[8] Muula AS, Prozesky HW, Mataya RH, Ikechebelu JI (2007). Prevalence of complications of male circumcision in Anglophone Africa: a systematic review. BMC Urol..

[9] Bailey RC, Egesah O, Rosenberg S (2008). Male circumcision for HIV prevention: a prospective study of complications in clinical and traditional settings in Bungoma, Kenya. Bull World Health Organ..

[10] Krieger JN, Bailey RC, Opeya J, Ayieko B, Opiyo F, Agot K, Parker C, Ndinya-Achola JO, Magoha GA, Moses S (2005). Adult male circumcision: Results of a standardized procedure in Kisumu District, Kenya. BJU Int..

[11] Opeya CJ, Ayieko BO, Kawango A Adult male circumcision in Kenya: safety and patient satisfaction (Abstract TuPeB4648)..

[12] Meredith H, Peters H, Viswanathan K, Rao D, Mashkoor A, Burnham G (2008). Client perceptions of the quality of primary care services in Afghanistan. International Journal for Quality in Healthcare..

[13] Wong Y, Leung M, Cheung L, Yam K, Yeoh K, Griffiths S (2011). A population-based survey using PPE-15: relationship of care aspects to patient satisfaction in Hong Kong. International Journal for Quality Health Care..

[14] Tung Y, Chang G (2009). Patient satisfaction with and recommendation of a primary care provider: associations of perceived quality and patient Education. International Journal for Quality Health Care..

[15] Woodside G, Nielsen L, Walters F, Muller D (1988). Preference segmentation of health care services: the old-fashioned, value conscious, affluent and professional want-it-all. Journal of Health Care Marketing..

[16] Gotlieb B (2000). Understanding the effects of nurses, patients' hospital rooms, and patients' perception of control on the perceived quality of a hospital. Health Marketing Quarterly..

[17] Herman-Roloff A, Otieno N, Agot K, Ndinya-Achola J, Bailey RC (2011). Acceptability of medical male circumcision among uncircumcised men in Kenya one year after the launch of the national male circumcision program. PLoS One..

[18] Mattson CL, Bailey RC, Muga R, Poulussen R, Onyango T (2005). Acceptability of male circumcision and predictors of circumcision preference among men and women in Nyanza Province, Kenya. AIDS care..

[19] Bailey RC, Muga R, Poulussen R, Abicht H (2002). The acceptability of male circumcision to reduce HIV infections in Nyanza Province, Kenya. AIDS care..

[20] Kim C, McEwen LN, Kerr EA, Piette JD, Chames MC, Ferrara A, Herman WH (2007). Preventive counseling among women with histories of gestational diabetes mellitus. Diabetes Care..

[21] Chinkonde JR, Sundby J, Martinson F (2009). The prevention of mother-to-child HIV transmission programme in Lilongwe, Malawi: why do so many women drop out?. Reproductive Health Matters..

[22] Duff P, Kipp W, Wild TC, Rubaale T, Okech-Ojony J (2010). Barriers to accessing highly active antiretroviral therapy by HIV-positive women attending an antenatal clinic in a regional hospital in western Uganda. Journal of the International AIDS Society..

[23] Kasenga F, Byass P, Emmelin M, Hurtig A (2009). The implications of policy changes on the uptake of a PMTCT programme in rural Malawi: first three years of experience. Global Health Action..

[24] Schoenfelder T, Klewer J, Kugler J (2011). Analysis of factors associated with patient satisfaction in ophthalmology: the influence of demographic data, visit characteristics and perceptions of received care. Journal of the College of Optometrists..

[25] Sekandi N, Makumbi E, Kasangaki A, Kizza B, Tugumisirize J, Nshimye E, Mbabali S, Peters H (2011). Patient Satisfaction with Services in Outpatient Clinics at Mulago hospital, Uganda. International Journal for Quality Health Care..

[26] Mattson L, Bailey C, Muga R (2005). Acceptability of male circumcision and predictors of circumcision preference among men and women in Nyanza Province, Kenya. AIDS care..

[27] Schoenfelder T, Klewer J, Kugler J (2011). Determinants of patient satisfaction: a study among 39 hospitals in an in-patient setting in Germany. International Journal for Quality in Health Care..

[28] Muhondwa E, Leshabari M, Mwangu M, Mbembati M, Ezekiel J (2008). Patient Satisfaction At The Muhimbili National Hospital In Dar Es Salaam, Tanzania. East African Journal of Public Health..

[29] Birhanu Z, Assefa T, Woldie M, Morankar S (2010). Determinants of satisfaction with healthcare provider interactions at health centers in Central Ethiopia: a cross sectional study. Biomedical Central Health Services Research..

